# Vitamin D intervention in preschoolers with viral-induced asthma (DIVA): a pilot randomised controlled trial

**DOI:** 10.1186/s13063-016-1483-1

**Published:** 2016-07-26

**Authors:** Megan E. Jensen, Genevieve Mailhot, Nathalie Alos, Elizabeth Rousseau, John H. White, Ali Khamessan, Francine M. Ducharme

**Affiliations:** 1 Clinical Research and Knowledge Transfer Unit on Childhood Asthma, Research Centre, Sainte-Justine University Health Centre, Montreal, Quebec Canada; 2 Department of Nutrition, University of Montreal, Montreal, QC Canada; 3 Department of Pediatrics, University of Montreal, Montreal, QC Canada; 4 Department of Physiology and Medicine, McGill University, Montreal, QC Canada; 5 Euro-Pharm International Canada, Montreal, QC Canada; 6 Departments of Pediatrics and of Social and Preventive Medicine and Clinical Research and Knowledge Transfer Unit on Childhood Asthma, Research Centre, Sainte-Justine University Health Centre, Université de Montréal, Montreal, QC Canada

**Keywords:** Asthma, Child, Diet, Feasibility study, Vitamin D, Paediatric, Randomised controlled trial

## Abstract

**Background:**

Trials in school-aged children suggest vitamin D supplementation reduces asthma exacerbations. Primary aim: to examine whether vitamin D_3_ (100,000 IU) rapidly raises serum 25-hydroxyvitamin D (25OHD) ≥75 nmol/L in asthmatic preschoolers.

**Methods:**

In a double-blind, randomised, placebo-controlled trial, preschool-aged children with asthma received 100,000 IU vitamin D_3_ (intervention) or placebo (control), followed by 400 IU vitamin D_3_ daily for 6 months. Serum 25OHD was measured at baseline, 10 days, 3 and 6 months. Outcomes included the group difference in 25OHD change from baseline at 3 months (Δ25OHD); the proportion of children with 25OHD ≥75 nmol/L at 3 months; the pattern in serum vitamin D over 6 months; the proportion of children with hypercalciuria at any time point (safety); and group rates for oral corticosteroids. Continuous outcomes were analysed using generalised linear mixed models and group rate ratios of events per child were assessed using a Poisson distribution model.

**Results:**

Twenty-two children were randomised (intervention:11; control:11) during winter. At 3 months, the group difference in Δ25OHD (7.2 nmol/L; 95 % CI: -13.7, 28.1) was not significant; yet, 100 % versus 54.5 % (intervention versus control) had serum 25OHD ≥75 nmol/L. There was a significant group difference in Δ25OHD at 10 days (110.3 nmol/L; 95 % CI: 64.0, 156.6). One child in each group had transient hypercalciuria at 10 days. Group oral corticosteroids rates were 0.82 and 1.18/child, intervention versus control (rate ratio = 0.68; 95 % CI: 0.30, 1.62; non-significant).

**Conclusions:**

Following 100,000 IU vitamin D_3_, all children reached serum 25OHD ≥75 nmol/L, compared with half who received placebo. Daily supplementation, sun exposure and insufficient power may explain the absence of a significant 3-month group difference in Δ25OHD. No clinically important alterations in bone metabolism biomarkers occurred. Group oral corticosteroid rates will inform sample size calculations for the larger trial. (NCT01999907, 25 November 2013).

**Electronic supplementary material:**

The online version of this article (doi:10.1186/s13063-016-1483-1) contains supplementary material, which is available to authorized users.

## Background

Preschoolers experience significant morbidity due to asthma and have the highest rate of emergency department visits attributable to asthma [1], with a notable proportion hospitalised following an acute care visit [2]. This is not surprising considering wheeze, the most common symptom associated with asthma in preschoolers [3], affects up to 50 % of children before age six [4]. More than 80 % of asthma exacerbations in children are triggered by viral upper respiratory tract infections (URTIs) [5]. URTIs occur at a rate of up to eight per year in preschoolers with recurrent wheeze or asthma [6, 7], the most common being rhinovirus [5]. It is therefore pertinent to explore preventive strategies to reduce the frequency and severity of viral-induced asthma exacerbations in preschool-aged children.

To date, pharmacological therapeutic avenues have predominantly been explored. Daily low-dose inhaled corticosteroids (ICS) and pre-emptive high-dose ICS are both effective in reducing the frequency of moderate to severe exacerbations requiring rescue oral corticosteroids [8, 9]. Uncertainties about the diagnosis and worry about side effects have limited the uptake of both strategies by physicians and/or parents. In paediatric observational studies, circulating 25-hydroxyvitamin D (25OHD) has been inversely associated with asthma severity, ICS dose, and the number of exacerbations requiring rescue oral corticosteroids or health care utilisation [10–12], as well as increased risk of viral URTIs [13]. In three placebo-controlled randomised trials of school-aged children with asthma, daily [14, 15] or monthly [16] vitamin D supplementation significantly reduced the rate of mild (requiring rescue short-acting β_2_-agonists, SABA) [14, 15] and moderate (requiring an acute care visit) [16] asthma exacerbations, with most exacerbations reportedly preceded by URTIs [14, 15]. Although it has not yet been investigated in preschool-aged children with recurrent viral-induced exacerbations, vitamin D supplementation appears promising to decrease the frequency of asthma exacerbations. Moreover, the use of a large dose administered by a health care professional, as opposed to daily supplementation, is likely to rapidly increase serum 25OHD levels to the desired target and remove the issue of non-compliance associated with patient-administered supplementation.

The aim of this pilot randomised controlled trial (RCT) was to investigate whether a single large dose of vitamin D_3_ effectively raises serum 25OHD to levels considered sufficient for potential extra-skeletal benefits (≥75 nmol/L [≥30 ng/mL]) [17, 18], in preschool-aged children with viral-induced asthma, compared with placebo. Second, we aimed to assess the feasibility of recruitment, intervention, procedures, and follow-up, and obtain pilot efficacy and safety data to inform the design of a large RCT.

## Methods

### Trial design

We conducted a 6-month randomised, parallel-group, double-blinded, placebo-controlled pilot trial of vitamin D_3_ (cholecalciferol) supplementation in preschool-aged children with viral-induced asthma. Patients were recruited in the asthma clinic, hospital wards, and emergency department of the Sainte-Justine University Health Centre, Montreal, QC, Canada, during the winter months, November 2013 to February 2014. The study was approved by the Sainte-Justine University Health Centre Research Ethics Board (#3760) and registered on ClinicalTrials.gov (NCT01999907, November 2013). Written informed consent was obtained from parents prior to their child’s participation.

### Participants

Children aged 1–5 years were eligible if they had: (i) physician-diagnosed asthma, based on clinical signs of airflow obstruction and reversibility [3, 19]; (ii) URTIs as the main exacerbation trigger, reported by parents; (iii) ≥4 parent-reported URTIs in the past 12 months; and (iv) ≥1 exacerbation requiring oral corticosteroids in the past 6 months or ≥2 in the past 12 months. Exclusion criteria included: extreme prematurity (<28 weeks’ gestation); high risk of vitamin D deficiency; other chronic respiratory disease; disordered calcium or vitamin D metabolism; oral medications interfering with vitamin D metabolism; or vitamin D supplementation greater than 1000 IU/day in the past 3 months.

### Interventions and procedures

Additional file 1 details the study design and schedule of procedures. At baseline, subjects received orally 2 mL of either 50,000 IU/mL vitamin D_3_ or identical placebo (Euro-Pharm International Canada, Montreal, QC, Canada). The dose was administered by the nurse, followed by 30 minutes of monitoring. The investigators initially believed it was unethical to not provide vitamin D to the control group, who were suspected to have low serum 25OHD levels at recruitment; thus, all participants, irrespective of group assignment, were provided with Pediavit D400 (Euro-Pharm International Canada, Montreal, QC, Canada) and instructed to take 1 mL daily (400 IU vitamin D_3_) during the study. Adherence was assessed by weighing the bottles before dispensation and at their return at each clinic visit.

Patient characteristics were documented at baseline, including skin type [20] and asthma control [21]. Non-fasting blood and urine samples were collected at baseline, 10 days (±3 days), 3 and 6 months (±0.5 months). With the exception of baseline samples, which were analysed immediately, serum aliquots preserved for vitamin D analysis were stored at -80 °C for batch analysis at the end of the study to maintain blinding. Total 25OHD (comprised of 25OHD_2_, 25OHD_3_ and 3-epimer-25OHD_3_) was analysed via tandem mass spectrometry [22]. Safety biomarkers were analysed immediately using standard laboratory processes.

Within 2 days of symptom onset (runny or congested nose, fever, or cough), parents collected a nasal secretion sample from the child’s nostril using a flocked mid-turbinate swab, which was then placed in a tube containing 1 mL Universal Transport Medium (Copan Italia, Brescia, Italy). The swab was transported by courier for storage at -80 °C before polymerase chain reaction (PCR) analysis [23] for 26 viruses (Laval University, Quebec City, QC, Canada). Parents also completed three questionnaires: the Canadian Acute Respiratory Illness and Flu Scale (CARIFS) [24]; the Asthma Flare-up Diary for Young Children (ADYC) [25]; and the Effects of an Asthma Flare-up on the Parents (ECAP) [26].

### Randomisation and blinding

Using computer-generated randomisation with variable block sizes of 2–4, participants were randomised 1:1 to the intervention or control group. Group assignment, recorded on a sequentially numbered list, was allocated by the Sainte-Justine Hospital Research Pharmacy, which held the randomisation code. To maintain blinding, the intervention and placebo dose were identical in colour, appearance, volume, taste, and packaging. All research personnel, physicians, nurses, participants and their parents were blinded to group allocation.

### Outcomes

The primary outcome was the mean group change in total serum 25OHD from baseline (Δ25OHD) to 3 months. Secondary outcomes were the group difference in the proportion of children with total 25OHD ≥75 nmol/L (30 ng/mL) at 3 months and in total 25OHD values over 6 months.

Exploratory outcomes included: (i) safety measures, namely the proportion of children with hypercalciuria (urinary calcium: creatinine ratio (Ca:Cr) >1.25 (1–2 years) and >1 (2–5 years) mmol/mmol) [27] at any time point, serum calcium, phosphorus, and alkaline phosphatase (ALP) [28]; and (ii) event rates for exacerbations requiring rescue oral corticosteroids (documented in medical and/or pharmacy records).

### Sample size

As prior information on the standard deviation for the change in total serum 25OHD was not available, the sample size was based on the expected proportion of patients with 25OHD ≥75 nmol/L (30 ng/mL) at 3 months, as reported in a previous study where 100 % of the intervention versus 55 % of the control subjects achieved a serum 25OHD level ≥75 nmol/L (30 ng/mL) [29]. Fifteen children per group were required to achieve 80 % power to detect a difference between the group proportions of 0.45, using a two-sided Mantel-Hansel test with alpha of 0.05. Allowing for a 10 % drop-out rate, we aimed to recruit 17 per group.

### Statistical methods

The change in serum 25OHD profile over the 6-month period was assessed using a generalised linear mixed model, which included as predictors: time, group, and a time*group interaction term. Covariates considered a priori for inclusion in the model were those with a distribution imbalance at baseline or a potential for confounding, such as baseline asthma classification (episodic versus persistent), asthma treatment, and dietary vitamin D. The mean group difference in Δ25OHD was reported with 95 % confidence interval (CI). Group rate ratios of events per child (RR) with 95 % CI were assessed using a Poisson distribution model. A two-sided *p* value ≤5 % was considered statistically significant. No adjustment for testing multiple outcomes was made. Analyses were conducted using SPSS® 21 (IBM SPSS Statistics, IBM Corp., Armonk, NY, USA) and SAS® 9.3 (SAS Institute, Cary, NC, USA).

## Results

Between November 2013 and February 2014, 85 children were screened: 45 were ineligible, primarily due to an insufficient number of oral corticosteroids (55.6 %) in the preceding year. Twenty-two children were randomised to the intervention (*N* = 11) or control group (*N* = 11) (Additional file 2). The trial was terminated before reaching the target sample size, as funding was received to commence the larger definitive trial. Retention in the intervention versus control group was 91 % vs. 100 % at 3 months, and 73 % vs. 91 % at 6 months.

Baseline characteristics were similar between the groups, with the exception of a higher prevalence of eczema, and a trend towards more persistent asthma and poorer asthma control in the control versus intervention group (Table 1). Baseline serum 25OHD was comparable between the intervention and control group (62.0 [50.0, 75.0] vs. 68.0 [50.0, 75.0] nmol/L), with the majority (*n* = 8, 72.7 %) in each group displaying baseline values <75 nmol/L (30 ng/mL); none were in the potentially rachitic range (<25 nmol/L [10 ng/mL]).

**Table 1:** Baseline subject characteristics

Characteristics	Intervention (*N* = 11)	Control (*N* = 11)
Age (years)	2.2 (1.9, 3.5)	3.1 (2.1, 3.9)
Male gender	4 (36.4 %)	3 (27.3 %)
Caucasian ethnicity^*^	5 (45.5 %)	8 (72.7 %)
Asthma control (Asthma Quiz for Kidz [20]), score	1.00 (0.00, 3.00)	4.00 (0.00, 6.00)
Asthma classification: *Persistent*	5 (45.5 %)	8 (72.7 %)
*Episodic*	6 (54.5 %)	3 (27.3 %)
Trigger of asthma: *Multitrigger*	3 (27.3 %)	4 (36.4 %)
*Unitrigger*	8 (72.7 %)	7 (63.6 %)
Eczema	8 (72.7 %)	2 (18.2 %)
Daycare attendance	9 (81.8 %)	11 (100 %)
Parent-reported URTIs in the past 12 months, number	6.0 (5.0, 12.0)	5.0 (4.0, 10.0)
Oral corticosteroid courses in the past 12 months, number	2.0 (1.0, 3.0)	2.0 (1.0, 3.0)
Influenza vaccine received	5 (45.5 %)	5 (45.5 %)
Preventer asthma medication: *Episodic ICS*	2 (18.2 %)	2 (18.2 %)
*Daily ICS ± anti-leukotrienes*	8 (72.7 %)	9 (81.8 %)

Results are reported as median (25 %, 75 %) or n (%)

*URTI* upper respiratory tract infection, *ICS*, inhaled corticosteroid

^*^Other ethnicities: Maghreb (9.1 %), Mixed (9.1 %), Latino (9.1 %), Arab (9.1 %), Asian (4.5 %)

Median daily dietary intake of vitamin D was markedly below the recommended 600 IU [30] in the intervention [356.0 (161.0, 510.0)] and control group [189.0 (166.0, 281.0)] at baseline. Dietary intake of vitamin D did not change significantly at 3 or 6 months in the intervention [274.0 (202.8, 601.5); 284.0 (157.0, 296.0)] or control group [198.0 (163.5, 283.5); 155.0 (134.0, 217.5)].

Adherence with the study bolus (100,000 IU vitamin D_3_/placebo) dose was 100 %. Most participants (65–80 %) returned the vitamin D bottles for weighing; median adherence was >92 % in both groups. The group difference in Δ25OHD was not statistically significant at 3 months (7.2 nmol/L; 95 % CI: -13.7, 28.1); however, 100 % of children in the intervention group, compared to 54.5 % of the control group, had vitamin D levels ≥75 nmol/L (*p* = 0.035). There was a statistically significant change in the mean total 25OHD over the 6 months (*p* ˂0.001), with a significant group, time, and group*time interaction effect (Additional file 3). Skin colour (*p* = 0.041) and the interaction between visit and asthma classification (*p* = 0.007) were significant variables in the model; conversely, parent-reported median daily sun exposure or season of measurement, which did not differ between groups, were not significant covariates. At 10 days, the within-group Δ25OHD was statistically significant for the intervention, but not the control group [132.5 (95 % CI: 100.4, 164.7) vs. 22.2 (-10.5, 55.0) nmol/L], with a significant between-group difference [110.3 (64.0, 156.6) nmol/L]. The within-group Δ25OHD was statistically significant at both 3 and 6 months in the intervention [27.1 (12.7, 41.4); 34.4 (21.7, 47.0) nmol/L] and control groups [19.8 (5.2, 34.5); 22.2 (10.5, 34.0) nmol/L]; but there were no between-group differences. The unadjusted values for serum 25OHD are presented in Additional file 4.

Two patients in the intervention group had an elevated total 25OHD (>225 nmol/L [90 ng/mL]) at 10 days (236 and 358 nmol/L); in both cases, the urinary Ca:Cr was normal. One child in the intervention group had hypercalciuria at 10 days, and one child in the control group had hypercalciuria at baseline and 10 days; none were associated with an elevated 25OHD value (196, 67 and 83 nmol/L, respectively) or hypercalcemia. There were several minor fluctuations in serum calcium, phosphorus and ALP, in both groups over the 6 months (Additional file 5); none were of clinical significance. No serious clinical or laboratory adverse health events occurred.

The rate of URTIs per patient month of follow-up did not differ between the intervention and control group [0.53 vs. 0.67; RR 0.74 (95 % CI: 0.46, 1.17)]; with 70.8 % (intervention) and 76.0 % (control) confirmed positive for at least one virus. The rate of acute-care visits [0.64 vs. 0.73; RR 0.88 (95 % CI: 0.32, 2.41)] and oral corticosteroids [0.82 vs. 1.18; RR 0.68 (95 %CI: 0.30, 1.62)] per child was not significantly different between the intervention and control group; one hospital admission occurred in the control group only.

## Discussion

This pilot RCT demonstrated the feasibility of an oral dose of 100,000 IU vitamin D_3_ to achieve vitamin D sufficiency at 3 months in all intervention patients, compared to about half of those allocated placebo. This observation did not translate into a statistically significant group difference in the change in total serum 25OHD from baseline to 3 months, although significantly more intervention children achieved vitamin D sufficiency at 3 months. Bone metabolism biomarkers provide preliminary data on the safety of the intervention, while the observed group rate of exacerbations requiring oral corticosteroids informs power calculations for the full-scale trial.

With approximately three-quarters of participants vitamin D-insufficient at baseline, 100,000 IU of vitamin D_3_ was effective in rapidly raising total serum 25OHD by 10 days and maintaining vitamin D sufficiency at 3 months in 100 % of the intervention group, compared to barely half of those assigned to the placebo. This rapid rise in serum 25OHD has previously been demonstrated in one infant [31] and one adolescent trial [32], 15 days following 100,000 and 200,000 IU, respectively, of oral vitamin D_3_. Concordant with a prior trial reporting 55 % of those receiving 400 IU daily vitamin D_3_ achieved 25OHD levels ≥75 nmol/L (30 ng/mL) at 3 months [29], our data suggest that, while daily 400 IU in addition to current dietary intake (approximately 200 IU) prevents the expected serum 25OHD decline in the fall and winter (with significant within-group increases at 3 and 6 months in the control group), it is insufficient to achieve vitamin D levels ≥75 nmol/L (30 ng/mL) in control patients. Yet, our main endpoint, group difference in the change in total 25OHD from baseline to 3 months, was not statistically significant. This non-separation of the groups was likely due to one, or a combination, of three main factors: (i) daily vitamin D_3_ supplementation in the control group; (ii) increased opportunity for ultraviolet B exposure [17], as more than half of the 3-month visits occurred in the spring; and (iii) our small sample size. In future trials, we would recommend the dose of 100,000 IU vitamin D_3_ be administered in early fall or winter, to pre-empt the expected seasonal decline in 25OHD and minimise the influence of sun exposure in spring and summer [17], and removal of daily vitamin D_3_ supplementation in the control group.

Although not powered to firmly conclude, our results contribute data supporting the safety of a dose of 100,000 IU vitamin D_3_ in preschool-aged children with asthma; there were no cases of potentially toxic 25OHD levels [18] or clinically significant changes in bone metabolism biomarkers. Of the five paediatric trials testing repeated oral doses of 100,000 IU vitamin D [31, 33–36], all but one measured circulating 25OHD [36]; just one trial reported two cases (0.3 %) of 25OHD >375 nmol/L (150 ng/mL) in a population of malnourished infants [36], but did not measure serum or urine bone metabolism biomarkers [35, 36], such that the clinical significance of the findings is unclear. Hence, only three of the five aforementioned trials provided additional safety data for a total of 15 infants [31] and 47 school-aged children and adolescents [33, 34]. Of these, one found ALP significantly decreased 9 months after study initiation in infants [31], and another reported one transient hypercalciuria event in the placebo group [34]; neither were clinically important alterations. Likewise, the few hypercalciuria events, documented in one subject of each group in our study, were transient. Occasional minor deviations from normal laboratory values were likely due to the non-fasting status of our patients, in contrast to a trial where subjects fasted [37]. While the administration of two spaced doses of 80,000–100,000 IU vitamin D every winter is recommended for all preschool-aged children in France [38], the evidence supporting the safety profile of this approach is weak and thus requires confirmatory data to be adopted in practice in North America. In future trials, we recommend the documentation of circulating 25OHD and bone metabolism biomarkers, including fasting urinary calcium:creatinine, in all subjects, with greater power to contribute data regarding the safety of a high-dose vitamin D_3_ oral bolus.

Our trial also aimed to provide health event rates to inform sample size calculations for the subsequent large-scale trial. The observed trend towards a 32 % reduction in the rate of exacerbations requiring oral corticosteroids with high-dose vitamin D_3_ is promising, but is not statistically significant. In three placebo-controlled RCTs of school-aged children with asthma, vitamin D supplementation has significantly reduced the rate of mild (requiring only rescue β_2_-agonists) to moderate (requiring emergency visits) asthma exacerbations by 64 % (RR: 0.36) with 500 IU/ day for 6 months [14], by 83 % (RR: 0.17) with 1200 IU/ day for 4 months [15], and by 53 % (RR: 0.47) with 60,000 IU monthly for 6 months [16]. Although the optimal delay between doses remains to be confirmed, repeat dosing in the context of a longer intervention period may be needed to increase the duration of vitamin D sufficiency, and thus maximise the likelihood of vitamin D supplementation benefit. Vitamin D may reduce asthma exacerbations via improving resistance to viral infections [39] (a main trigger of asthma exacerbations) [5], modulating the inflammatory response [40], either directly or indirectly by improving response to corticosteroid maintenance medications, or relaxation of bronchial smooth muscle [41]. The observed trend towards fewer exacerbations warrants further investigation in trials adequately powered to detect a statistically significant group difference in this important health outcome for children with asthma.

This study has several notable aspects, namely, dose administration by research personnel in the routine clinical setting, which achieved 100 % compliance and avoided the potential non-compliance associated with self-administered daily supplementation [15, 29]. Importantly, there was no clinically important change in dietary vitamin D intake over the 6-month period, indicating no evidence of Hawthorne bias; that is, participation in the study did not lead to higher dietary intake of vitamin D in either group. In addition, we used the gold-standard method, tandem mass spectrophotometry, to quantify serum vitamin D_3_, including epimer-D_3_. The documentation of several bone metabolism biomarkers offers preliminary data towards the safety of this supplementation dose.

We acknowledge several limitations to this trial, namely, being underpowered for our specified primary outcome, which possibly impaired our ability to firmly conclude on our main outcome. However, we detected a statistically significant difference in the proportion of children with vitamin D sufficiency, our secondary outcome, from which our sample size was calculated. It is also possible that the period during which children maintained vitamin D sufficiency was not sufficient to allow the proposed effects of vitamin D to be observed; thus, a longer intervention, with repeat dosing, may be warranted. As the administration of daily 400 IU in both groups probably prevented group separation, future studies could consider removal of daily supplementation in the control group alone to maximise group separation. Indeed, a recent review suggests that daily vitamin D supplementation may be more effective than bolus dosing in reducing respiratory tract infections [42], and the potential for adverse effects of rapid shifts in local tissue 1,25OHD levels induced by bolus dosing has been proposed [43]. This has led to some suggestion that more regular supplementation of vitamin D could be a preferred regimen, e.g. daily or weekly, with or without bolus dosing, to more smoothly elevate and maintain serum 25OHD; however, one must consider in their design the time it would take to reach the target level of 75 nmol/L (intervention would need to be started months prior to the desired period for anticipated effect) and the compliance issues with placing the burden of supplementation on the parent/carer. Regardless of treatment regimen, the evidence to date suggests that vitamin D supplementation may be more effective in improving asthma outcomes in the paediatric population as opposed to adults [44], and ours is the first trial to examine the use of a bolus vitamin D dose in children with asthma. In terms of compliance and rapidly attaining a serum 25OHD level >75 nmol/L, the bolus dose appears ideal.

Our group included both children with persistent and episodic asthma. One may hypothesise that the response to vitamin D potentially differs between these groups. However, even amongst asthma specialists, there is notable intra-patient and between-physician variability in classification [45, 46], due to third-party reporting of symptoms and the inability to obtain standard lung function tests in preschoolers; thus, we acknowledge that the accuracy of this classification is debatable. We also acknowledge that some would hesitate to definitively diagnose asthma in children <2 years of age, instead classing this group as ‘early wheezers’; however, we applied the Canadian guidelines for asthma diagnosis in preschoolers (i.e. observed obstruction and reversibility) for inclusion in the study [19]. Although our small sample size did not allow stratification of analyses to examine whether vitamin D response differed by age group or asthma classification, this is an interesting consideration for future trials.

## Conclusions

This pilot study confirmed the feasibility and effectiveness of the intervention to rapidly raise total serum 25OHD and correct vitamin D insufficiency; however, our study was underpowered to detect a significant group difference in Δ25OHD at 3 months. With no cases of a toxic serum 25OHD value or clinically important alteration in key biomarkers, our data inform, but do not prove, the safety of the intervention. Our results provide promising data with a non-significant trend towards a reduction in the rate of rescue oral corticosteroids in the intervention group, which underlines the need for a large-scale trial. The ideal design to test the efficacy of high-dose vitamin D_3_ should include the administration of the dose early in the fall or winter, without concurrent daily vitamin D supplementation.

## Abbreviations

25OHD, 25-hydroxyvitamin D; ADYC, Asthma Flare-up Diary for Young Children; ALP, alkaline phosphatase; Ca:Cr, calcium: creatinine ratio; CARIFS, Canadian Acute Respiratory Illness and Flu Scale; CI, confidence interval; ECAP, Effects of an Asthma Flare-up on the Parents; ICS, inhaled corticosteroids; PCR, polymerase chain reaction; RCT, randomised controlled trial; RR, rate ratio; SABA, short-acting β_2_-agonists; URTI, upper respiratory tract infection

## Additional files

Additional file 1: Study design and procedures. ^*^Asthma Quiz for Kidz [20], a 6-item questionnaire, where a score of two or more indicates poor asthma control. ^‡^A 3-item questionnaire, developed ad hoc. ^∫^A visual paediatric tool, developed ad hoc, adapted from the validated adult Fitzpatrick scale [19]. ^∫^Food checklists developed by dietitian (MEJ). ^¶^Asthma Flare-up Diary for Young Children [24]; Canadian Acute Respiratory Infection and Flu Scale [23]; and Effect of a Young Child’s Asthma Flare-up on the Parent questionnaires [25]. ^||^URTI, upper respiratory tract infection. ^**^ALP, alkaline phosphatase. (PDF 73 kb) Additional file 2: Patient selection and retention. The number of children included at screening, randomisation, follow-up and analysis are presented for the 6-month study. ^*^Children had less than one rescue oral corticosteroid course in the past 6 months, or less than two in the previous 12 months. ^†^Children had less than four parent-reported upper respiratory tract infections (URTIs) in the past 12 months. ^‡^Lost to follow-up before 3-month visit. ^∫^Withdrew before 6-month visit (*n* = 1 relocation; *n* = 2 protocol burden). (TIF 61 kb) Additional file 3: Serum 25-hydroxy vitamin D levels over 6 months. The estimated marginal means for total serum 25-hydroxy vitamin D (25OHD) are presented, by group, over the 6-month study period. Error bars represent the 95 % confidence interval around the mean. The *dotted line* at 75 nmol/L (30 ng/mL) on the y-axis indicates the cut point between proposed vitamin D sufficiency and insufficiency. (TIF 189 kb) Additional file 4: Unadjusted serum 25-hydroxy vitamin D values over 6 months. The unadjusted values for total serum 25-hydroxy vitamin D (25OHD) are presented, by group, over the 6-month study period. Individual data points are represented, with the mean indicated by the *straight line*. The *dotted line* at 75 nmol/ L (30 ng/mL) on the y-axis indicates the cut point between proposed vitamin D sufficiency and insufficiency. (TIF 259 kb) Additional file 5: Safety biomarkers over 6 months. The non-fasting values for: (A) urinary calcium: creatinine, (B) serum calcium, (C) serum alkaline phosphatase, and (D) serum phosphorous, are presented as box-and-whiskers (Tukey) plots, by group, over the 6-month study period. Outliers are represented by ‘^*^’. Reference laboratory values for given age groups are presented by the *dotted* and *dashed lines* in each graph: [25] A *Dotted line* represents upper limit (1.25) for ages 1–2 years, and *dashed line* represents upper limit (1.00) for ages 2–5 years. B *Dashed lines* represent upper (2.62) and lower (2.3) limit for ages 0–18 years. C *Dashed lines* represent upper (369) and lower (156) limit for ages 1–9 years. D *Dotted line* represents upper (1.47) and lower limit (1.03) for ages 5–12 years, and *dashed line* represents upper (1.7) and lower (1.08) limit for ages 1–4 years. (TIF 584 kb)
