# Supplementary figures and images for: Vitamin D intervention in preschoolers with viral-induced asthma (DIVA): a pilot randomised controlled trial
Source: Trials. 2016 Jul 26;17:353. doi: 10.1186/s13063-016-1483-1 (PMC4960871; doi:10.1186/s13063-016-1483-1)

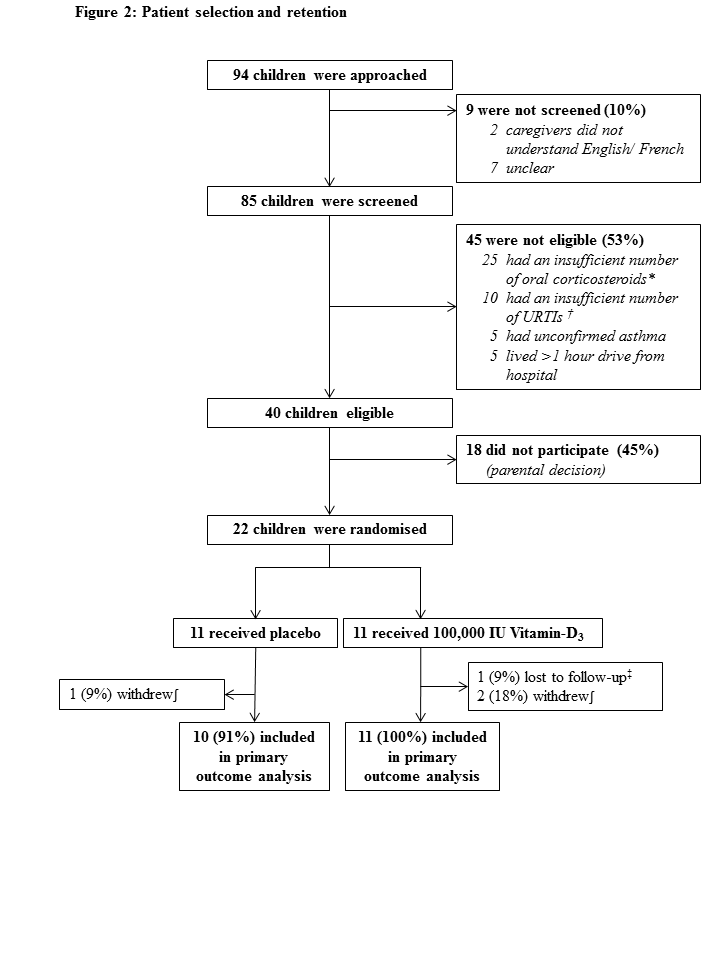

Supplement: Additional file 2: — Patient selection and retention. The number of children included at screening, randomisation, follow-up and analysis are presented for the 6-month study. *Children had less than one rescue oral corticosteroid course in the past 6 months, or less than two in the previous 12 months. †Children had less than four parent-reported upper respiratory tract infections (URTIs) in the past 12 months. ‡Lost to follow-up before 3-month visit. ∫Withdrew before 6-month visit (n = 1 relocation; n = 2 protocol burden). (TIF 61 kb) [file 13063_2016_1483_MOESM2_ESM.tif]
