# Supplementary material for: Vitamin D intervention in preschoolers with viral-induced asthma (DIVA): a pilot randomised controlled trial
Source: Trials. 2016 Jul 26;17:353. doi: 10.1186/s13063-016-1483-1 (PMC4960871; doi:10.1186/s13063-016-1483-1)
Supplement: Additional file 1: — Study design and procedures. *Asthma Quiz for Kidz [20], a 6-item questionnaire, where a score of two or more indicates poor asthma control. ‡A 3-item questionnaire, developed ad hoc. ∫A visual paediatric tool, developed ad hoc, adapted from the validated adult Fitzpatrick scale [19]. ∫Food checklists developed by dietitian (MEJ). ¶Asthma Flare-up Diary for Young Children [24]; Canadian Acute Respiratory Infection and Flu Scale [23]; and Effect of a Young Child’s Asthma Flare-up on the Parent questionnaires [25]. ||URTI, upper respiratory tract infection. **ALP, alkaline phosphatase. (PDF 73 kb) [file 13063_2016_1483_MOESM1_ESM.pdf]

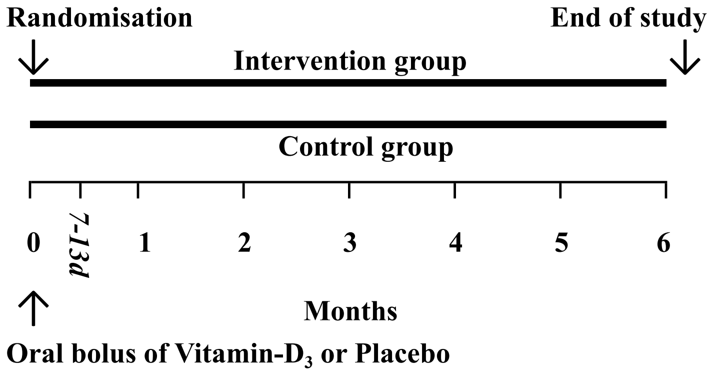

**Clinic Visits**								
Non-fasting blood and urine samples	✓				✓			✓
Asthma status and control*	✓				✓			✓
Sun exposure questionnaire†	✓				✓			✓
Skin colour (Fitzpatrick scale)‡	✓							
Dietary intake (vitamin D, Calcium) ʃ	✓				✓			✓
Exchange of daily vitamin D bottles - compliance	✓				✓			✓
Dispense / exchange of questionnaires¶	✓				✓			✓
Dispensation of nasal swabs	✓				✓			
Adverse health events	✓				✓			✓
URTIs,‖ exacerbations, medications, acute care visits or hospitalisations	✓				✓			✓
**Home visit**								
Non-fasting blood and urine samples		✓						
**Telephone Contacts**								
Adverse health events		✓	✓	✓	✓	✓	✓	✓
URTIs,‖ exacerbations, medications, acute care visits or hospitalisations		✓	✓	✓	✓	✓	✓	✓
Daily vitamin D supplement compliance		✓	✓	✓	✓	✓	✓	✓
**Laboratory analyses***								
Urinary calcium: creatinine ratio	✓	✓			✓			✓
Serum calcium, phosphorus, and ALP**	✓	✓			✓			✓
Serum 25-hydroxy vitamin D	✓	✓			✓			✓
